# Apolipoproteins in Chronic Kidney Disease and Kidney Transplant: A Long Unfinished Story

**DOI:** 10.3390/ijms26199664

**Published:** 2025-10-03

**Authors:** Carmine Secondulfo, Carmine Izzo, Nicoletta Vecchione, Gianmarco Minelli, Dora Russo, Donatella Russo, Rossella Barra, Gabriella Molinaro, Luca Apicella, Candida Iacuzzo, Antonio Pisani, Sarah Hamzeh, Maria Amicone, Massimo Cirillo, Giancarlo Bilancio

**Affiliations:** 1 Department of Medicine, Surgery and Dentistry “Scuola Medica Salernitana”, University of Salerno, 84081 Baronissi, Italy; csecondulfo@unisa.it (C.S.); cizzo@unisa.it (C.I.); mcirillo@unisa.it (M.C.); 2 Department of Public Health, University of Naples “Federico II”, 80131 Naples, Italy; nicolettavecchione@gmail.com (N.V.); gianmarcominelli@gmail.com (G.M.); donatellarusso0891@gmail.com (D.R.); rossella2_barra@libero.it (R.B.); gabri.molinaro@libero.it (G.M.); antonio.pisani13@gmail.com (A.P.); sarahhamzeh1@gmail.com (S.H.); ma.amicone.90@gmail.com (M.A.); 3 Unit of Nephrology, Dialysis and Transplant, Salerno University Hospital “San Giovanni di Dio e Ruggid’Aragona”, 84131 Salerno, Italy; luca.apicella@sangiovannieruggi.it (L.A.); candida.iacuzzo@sangiovannieruggi.it (C.I.)

**Keywords:** dyslipidemia, apolipoprotein, PCSK9, chronic kidney disease, kidney transplant

## Abstract

Chronic kidney disease (CKD) is a growing global health burden, strongly associated with cardiovascular disease, the leading cause of mortality in this population. Dyslipidemia is a key metabolic abnormality in CKD, but traditional lipid measures such as total cholesterol, LDL cholesterol, HDL cholesterol, and triglycerides often fail to capture the complexity of lipid disturbances in CKD and after kidney transplantation. Apolipoproteins have emerged as more reliable markers of cardiovascular and renal risk. Elevated apolipoprotein B (ApoB), reduced apolipoprotein A1 (ApoA1), and a higher ApoB/ApoA1 ratio are linked to CKD progression, cardiovascular events, and post-transplant complications, including post-transplant diabetes mellitus. Lipoprotein(a), a genetically determined atherogenic lipoprotein, accumulates in CKD due to impaired clearance and further increases cardiovascular risk. Other apolipoproteins, such as APOL1 and APOE, modulate CKD susceptibility through lipid-dependent and independent mechanisms. In addition, proprotein convertase subtilisin/kexin type 9 (PCSK9) has been identified as an important regulator of lipid metabolism, and PCSK9 inhibitors may represent a promising therapeutic option, though evidence in advanced CKD and transplant recipients is still limited, especially regarding their effects on apolipoproteins. This review summarizes current evidence on apolipoproteins and PCSK9 in CKD and transplantation, with attention to their potential as biomarkers and therapeutic targets.

## 1. Introduction

Chronic kidney disease (CKD) is a prevalent global health condition associated with a substantial disease burden and significant morbidity. In 2017, an estimated 697.5 million cases were recorded worldwide, corresponding to a global prevalence of 9.1%. That same year, CKD was responsible for 35.8 million disability-adjusted life years and 1.2 million deaths [1]. Despite being both preventable and treatable, CKD has affected an increasingly large portion of the population. Between 1990 and 2017, its prevalence rose by 29.3%, and its all-age mortality rate increased by 41.5% [1]. This upward trend aligns with recent projections indicating that CKD may become the fifth leading cause of death globally [2].

CKD is a multifactorial disorder, in which both genetic predisposition and modifiable risk factors contribute to its development. In addition to inherited components [3], major contributors include diabetes, hypertension, and dyslipidemia, and particularly elevated triglyceride levels, increased serum low-density lipoprotein (LDL) cholesterol and reduced concentrations of high-density lipoprotein (HDL) cholesterol [4,5]. Metabolic abnormalities are recognized risk factors for the progression of chronic kidney disease [6], and among these, dyslipidemia represents a major component [7], as it may contribute to renal lipid accumulation and exacerbate both glomerular and tubulointerstitial injury through mechanisms involving inflammation and oxidative stress [8]. Consequently, alterations in lipid levels are considered important indicators of declining renal function. Lipoproteins and their components could play a role as both genetic and non-hereditary factors [9,10] involved in risk of developing and worsening CKD.

The assessment of dyslipidemia in both the general population and individuals with chronic kidney disease or end-stage renal disease (ESRD) commonly relies on measurements of total cholesterol, low-density lipoprotein cholesterol, high-density lipoprotein cholesterol, and triglycerides, primarily for cardiovascular risk evaluation; it is worth noting individuals with CKD face a markedly elevated risk of cardiovascular events. Approximately 50% of patients with CKD stages 4 to 5 have established cardiovascular disease (CVD). Cardiovascular mortality accounts for an estimated 40% to 50% of all deaths in patients with advanced CKD and ESRD, in contrast to a rate of 26% among individuals with normal kidney function [11].

This higher susceptibility to CVD suggests the need for a more intensive evaluation of cardiovascular health and risk stratification in this population. In addition to the aforementioned conventional markers, apolipoproteins have also been proposed as potential biomarkers to further extend the assessment of renal patients.

This review aims to provide a comprehensive overview of the existing body of knowledge on the topic, tracing the historical development of key concepts while integrating recent findings. Emerging pharmacological advances, such as Proprotein Convertase Subtilisin/Kexin type 9 (PCSK9) inhibitors, have renewed interest in this field, raising important questions for future investigation. By revisiting established evidence in light of these new perspectives, this work seeks to highlight potential directions for further research and clinical application. Embase, PubMed and Cochrane databases have been searched, in the period from June to July 2025, for the most recent or well-established high-quality evidence for the role of apolipoproteins, lipoproteins and PCSK9 in chronic kidney disease, together with consensus statements and international guidelines on the management of lipid-related anomalies in CKD and kidney transplantation.

## 2. Apolipoprotein A and B

Apolipoproteins are the proteic components that bind lipid to form lipoproteins.

Apolipoprotein A1 (ApoA1) is the key component of HDL [12], while Apolipoprotein B (ApoB) is structural for LDL [13].

ApoB is crucial for the atherogenic properties of LDLs. Although less abundant, other ApoB-containing lipoproteins exhibit markedly greater atherogenic potential per particle compared to LDL. While they likely contribute to atherogenesis through mechanisms similar to those of LDL, they may also trigger additional pathogenic pathways [13].

In patients with ESRD, conditions such as hypertriglyceridemia, malnutrition, and metabolic disturbances often result in paradoxically “normal” levels of LDL, while apolipoprotein B (ApoB) concentrations remain elevated due to increased production [14,15]. This imbalance favors the predominance of small, dense LDL particles (type B phenotype), which are highly atherogenic and play a central role in the development of atherosclerosis [16,17]. Elevated ApoB levels have also been observed in pre-dialysis and peritoneal dialysis (PD) patients, whereas in hemodialysis (HD) patients, plasma concentrations are generally within the normal range [18]. Higher ApoB levels are correlated to increased risk of atherosclerotic vascular events in patients with CKD [19]. Conversely, higher serum ApoA1 concentrations have been associated with a lower prevalence of chronic kidney disease and with higher estimated glomerular filtration rate (eGFR) [20].

Current evidences suggest that serum ApoB represents a more reliable biomarker than traditional lipid parameters for the diagnosis and prognosis of cardiovascular disease [21,22], as well as for monitoring the efficacy of lipid-lowering therapies. Moreover, studies on Chinese population confirmed that serum ApoB has been shown to exhibit the strongest correlation with CKD. Furthermore, elevated ApoB levels may precede the onset of CKD, suggesting that monitoring and lowering ApoB concentrations could represent a potential strategy for the prevention and management of CKD [23].

ApoB/ApoA1 ratio is currently regarded as a better serum marker for cardiovascular risk than HDL/LDL ratio [24,25], both in the general population and prevalent dialysis patients [26,27]. Furthermore, a higher apolipoprotein B to apolipoprotein A1 ratio has been associated with the progression of CKD [28]. In patients with immunoglobulin A nephropathy, Lundberg et al. reported that an elevated ApoB/ApoA1 ratio was significantly correlated with an increased risk of developing end-stage renal disease (ESRD) [29].

In kidney transplant recipients, the prognostic role of ApoA1 and ApoB is currently not well described. The post-transplant state is characterized by impaired triglyceride clearance [30]. Since ApoB is an essential structural component of very-low-density lipoprotein cholesterol, intermediate-density lipoprotein cholesterol, and low-density lipoprotein cholesterol, its concentrations may provide a more accurate measure of the hypertriglyceridemia burden associated with diabetes and may therefore serve as an indicator of a prediabetic state. Moreover, overproduction of ApoB has been shown to contribute to the development of insulin resistance [31]. Accordingly, the current literature suggests that ApoB/ApoA1 ratio is an effective predictor of late post-transplant diabetes mellitus (PTDM) development, and that could be more useful than traditional serum lipid screening [32,33].

## 3. Lipoprotein (a)

Lipoprotein(a) [Lp(a)] is considered one of the strongest genetically determined risk factors for cardiovascular disease (CVD). It is synthesized in the liver and consists of a low-density lipoprotein (LDL) particle [34] bound to an additional apolipoprotein, apolipoprotein(a) [apo(a)].

Although the kidney does not directly regulate lipoprotein synthesis or catabolism, CKD induces both quantitative and qualitative changes in plasma lipoproteins, leading to an atherogenic profile that may also accelerate renal disease progression [35,36,37]. Dyslipidemia in CKD is characterized by elevated triglycerides, reduced HDL cholesterol, normal or slightly decreased LDL cholesterol, and an accumulation of small, dense LDL particles, intermediate-density lipoproteins (IDL), and very-low-density lipoproteins (VLDL) [9,10,11]. These alterations are often compounded by increased levels of apolipoprotein B (apoB) and lipoprotein(a) [Lp(a)], with a concurrent reduction in apolipoproteins AI and AII [38,39].

The elevated levels of Lp(a) observed in CKD appear to be influenced by reduced glomerular filtration rate (GFR), supporting the hypothesis that renal function plays a role in its catabolism [39,40,41,42,43]. Lp(a) is a genetically regulated lipoprotein with known atherogenic and pro-thrombotic properties, and its accumulation in CKD may be a key contributor to the heightened cardiovascular risk in this setting [41,43]. Studies suggest that the increase in Lp(a) in CKD is not entirely due to synthesis but also to a diminished clearance, possibly through renal catabolic pathways [43]. In individuals with mild-to-severe CKD, both elevated Lp(a) concentrations and genetic determinants of Lp(a) levels have been shown to be significantly associated with cardiovascular disease (CVD) at baseline and during follow-up, independently of traditional risk factors [44].

In nephrotic syndrome, a distinct form of glomerular pathology, lipoprotein abnormalities are further exacerbated. There is a marked increase in LDL cholesterol, triglycerides, and all apoB-containing lipoproteins, including Lp(a), due to both enhanced hepatic synthesis and impaired clearance mechanisms [45,46,47]. Upregulation of PCSK9 in this context leads to downregulation of LDL receptors, contributing to hypercholesterolemia [48]. Additionally, Lp(a) levels are markedly increased, likely as a response to hypoalbuminemia and increased apoB production [49,50].

Lipoprotein profiles also vary depending on the modality of renal replacement therapy (Table 1). Hemodialysis (HD) patients typically present with hypertriglyceridemia, low HDL cholesterol, and elevated apoB levels, while patients on continuous ambulatory peritoneal dialysis (CAPD) tend to exhibit higher total and LDL cholesterol levels, likely due to glucose absorption from dialysate and resultant hepatic lipogenesis [51,52,53,54]. Lp(a) levels are elevated in both HD and CAPD patients, approximately threefold compared to those with less advanced CKD [51]. Acute hemodialysis sessions, through heparin administration, transiently reduce triglycerides and increase HDL cholesterol due to lipoprotein lipase (LPL) activation, although this is often followed by the generation of atherogenic remnant particles post-dialysis [55].

Following successful kidney transplantation, partial normalization of the lipid profile may occur. Triglyceride levels generally decrease, while total and HDL cholesterol levels increase [57,58]. Lp(a) levels tend to decline modestly, though not uniformly, and remain elevated in some cases despite improved renal function [59,60]. The post-transplant lipid profile is also influenced by immunosuppressive therapy, particularly corticosteroids and calcineurin inhibitors, which can promote hypercholesterolemia and impair HDL functionality [61]. In KTRs, combination therapy with statins and ezetimibe has been shown to achieve greater lipid-lowering effects, while PCSK9 inhibitors have been associated with a reduced incidence of adverse events and lower all-cause mortality. These findings indicate high efficacy and safety, supporting the potential for broader clinical application [61].

CKD and renal replacement therapies are associated with complex and heterogeneous lipoprotein abnormalities. These include increased levels of atherogenic particles such as small dense LDL, Lp(a), and triglyceride-rich remnants, along with impaired HDL composition and function. Such disturbances are influenced by both disease severity and treatment modality, and they persist, though variably, following kidney transplantation. Given their implications for cardiovascular morbidity and mortality, these abnormalities warrant careful monitoring and, where appropriate, targeted therapeutic intervention.

## 4. Other Apolipoproteins in CKD and Kidney Transplant

Beyond ApoA1, ApoB, and Lp(a), several other apolipoproteins have been implicated in the pathophysiology of chronic kidney disease (CKD) and kidney transplantation. These proteins, although less extensively studied, may provide additional insight into the genetic and metabolic determinants of disease risk, progression, and outcomes. Unlike classical apolipoproteins involved in lipid transport, such as ApoA1 and ApoB, apolipoprotein L1 (APOL1) and apolipoprotein E (APOE) exert more complex and diverse biological effects that extend beyond lipid metabolism. Variants in the APOL1 gene, for example, are strongly associated with increased susceptibility to CKD and accelerated graft loss in individuals of African ancestry, reflecting a unique intersection of evolutionary adaptation and renal vulnerability. Similarly, common APOE allelic variants (ε2, ε3, and ε4) have been shown to influence renal function, diabetic kidney disease progression, and cardiovascular risk, underscoring the dual metabolic and non-metabolic roles of apolipoproteins in kidney health.

In kidney transplant recipients, the relevance of these apolipoproteins is particularly noteworthy. Genetic predisposition linked to APOL1 and APOE may help explain inter-individual variability in transplant outcomes, complementing the well-established roles of immune and non-immune risk factors. Moreover, emerging mechanistic studies suggest that the pathogenic effects of these variants may involve isoform-specific cellular pathways, such as podocyte injury, mesangial proliferation, or altered vascular function, which are only partially dependent on lipid disturbances. Taken together, the study of “non-classical” apolipoproteins provides an important opportunity to expand the understanding of CKD pathophysiology and transplant outcomes, while offering potential for new biomarkers and therapeutic targets.

In the following sections, particular attention will be devoted to APOL1 and APOE, two apolipoproteins that illustrate how genetic variability can shape both renal disease susceptibility and transplant outcomes, through mechanisms that not only intersect with but also extend beyond lipid metabolism, thereby highlighting their relevance for future research, risk stratification, and the development of personalized therapeutic approaches in CKD and kidney transplantation.

### 4.1. Apolipoprotein L1

Apolipoprotein L1 (APOL1) genes mutation are known to confer, in people of African ancestry, a selective resistance to infection from trypanosome, as they can act as an ion channel with increased activity in trypanosome [62,63]. The same variants, however, are known to cause increased risk of chronic kidney disease. This effect is due to the same mechanism on podocytes, thus altering glomerular permeability [64]. The presence of two APOL1 risk variants (APOL1 RRV) confers a higher risk of developing chronic kidney disease compared to a single variant [65,66], but the development of CKD is not inevitable.

This increased risk of CKD is not associated with cardiovascular disease, thus suggesting an independent effect on kidney health by APOL1 [67]. Notably, APOL1 risk variants have not been consistently associated with an increased prevalence or severity of diabetic kidney disease (DKD) progression, despite DKD being the most common cause of end-stage kidney disease [1]. Although this apparent discrepancy remains unresolved, its clarification may offer valuable mechanistic and therapeutic insights. Activation of the renin–angiotensin-aldosterone system (RAAS) is known to play a central role in the development and progression of DKD. Recent in vitro and in vivo studies have also demonstrated that RAAS activation contributes to kidney cell injury in experimental models of APOL1-mediated kidney disease (AMKD). Both high glucose levels and the presence of APOL1 RRVs have been shown to increase podocyte expression of miR-193a, a recognized mediator of glomerulosclerosis, including idiopathic focal segmental glomerulosclerosis (FSGS) and DKD [68,69]. It has been hypothesized that RAAS activation and/or elevated miR-193a expression in the diabetic milieu may already create an environment maximally conducive to kidney cell injury, rendering the additional impact of APOL1 RRVs negligible [70].

In the United States, living kidney donors carrying high-risk APOL1 genotypes are more likely to develop advanced chronic kidney disease after donation [71]. Additionally, transplantation of kidneys from deceased donors with high-risk APOL1 genotypes is associated with faster graft failure [72,73]. APOL1 genotyping may be considered as one of several factors in the assessment of organ suitability or in the evaluation of risk for potential living kidney donors. Such data could help inform the balance between improved risk stratification for African American donors and the potential limitation of access to kidney transplantation [74].

Currently, no effective therapy is available for treatment of APOL1-mediated kidney disease, but there are ongoing phase 2 and 3 trials involving APOL1 small molecule inhibitors, APOL1 antisense oligonucleotides, and JAK/STAT pathway blockers [75,76,77].

### 4.2. Apolipoprotein E

Apolipoprotein E gene (APOE) presents three common different variants, called alleles ε2, ε3, and ε4. They are expressed in the kidney and differentially modulate lipoprotein metabolism [78].

Growing evidence suggests that apolipoprotein E (APOE) alleles have differential effects on kidney disease risk, particularly in individuals with diabetes. Several studies have shown that the ε2 allele is associated with an increased risk of diabetic nephropathy, both in type 1 and type 2 diabetes, as well as worse renal function in small case–control cohorts [79,80,81,82,83,84,85,86]. In contrast, the ε4 allele appears to be protective, as ε4 carriers with diabetes exhibit better renal function and a lower risk of developing diabetic nephropathy [14,17,21]. When later stages of kidney disease were assessed, such as end-stage renal disease (ESRD), ε4 was associated with a reduced risk [87,88,89], whereas ε2 was linked to an increased risk in at least one study [89].

**Figure 1:**
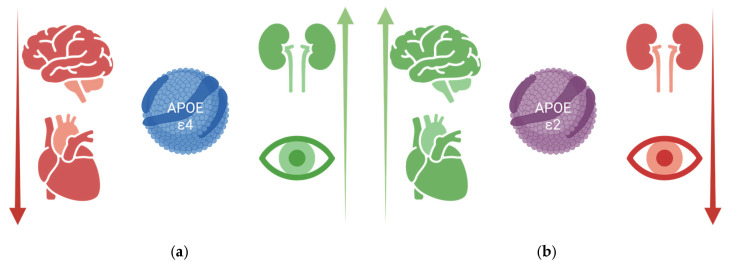
Schematic representation of ApoE alleles risk association with Alzhemier’s disease, coronary heart disease, diabetic nephropathy and age-related maculopathy. (**a**) Risk association for allele ε4 mutation (**b**) Risk association for allele ε2 mutation. Created in BioRender. Bilancio, G. (2025) https://BioRender.com.

These allelic effects on diabetic kidney disease mirror APOE associations observed in age-related maculopathy [90] but contrast with those seen in coronary heart disease (CHD) and Alzheimer’s disease [78,91] (Figure 1). While ε4 is a well-established genetic risk factor for Alzheimer’s disease and, to a lesser extent, for CHD [92], it has been associated with a favorable lipid profile, including higher levels of high-density lipoprotein and lower levels of triglycerides [78], which may contribute to reduced CKD risk [5]. Conversely, the ε2 allele is linked to type III hyperlipoproteinemia and elevated triglyceride levels due to delayed clearance [78], both of which are associated with increased CKD risk [5,93].

In addition to its effects on lipid metabolism, APOE may influence kidney disease progression through isoform-specific biological mechanisms, including differential modulation of vascular smooth muscle function and mesangial cell proliferation [94,95].

Taken together, the evidence on APOL1 and APOE illustrates how apolipoproteins can influence kidney disease and transplantation not only through lipid-related mechanisms but also via genetic and cellular pathways. Building on this broader view of lipid dysregulation, attention has increasingly shifted toward proprotein convertase subtilisin/kexin type 9 (PCSK9), a critical modulator of LDL receptor turnover, which represents both a marker of dyslipidemia and an emerging therapeutic target in CKD and kidney transplantation.

## 5. PCSK9

Proprotein convertase subtilisin/kexin type 9 (PCSK9) has emerged as a pivotal regulator of cholesterol homeostasis, acting primarily through the degradation of hepatic LDL receptors and thereby limiting the clearance of circulating ApoB-containing lipoproteins. While originally studied in the context of familial hypercholesterolemia and cardiovascular disease, PCSK9 has more recently gained attention in chronic kidney disease and kidney transplantation, where disturbances of lipid metabolism are frequent, multifactorial, and tightly linked to adverse cardiovascular outcomes. In kidney transplant recipients, dyslipidemia is further aggravated by immunosuppressive therapy and metabolic complications, raising questions about whether PCSK9 plays a role in post-transplant lipid disturbances and cardiovascular risk. Although data in this specific population remain limited, the proven efficacy and safety of PCSK9 inhibitors in the general population, particularly when added to statins and ezetimibe, highlight the therapeutic potential of targeting PCSK9 in CKD and transplantation.

While circulating PCSK9 concentrations do not appear to correlate directly with glomerular filtration rate, studies have shown that PCSK9 participates in the abnormal metabolism of triglyceride-rich lipoproteins, which are characteristically elevated in CKD patients [96].

This observation is consistent with the broader picture of CKD-associated dyslipidemia, which is marked not only by quantitative but also qualitative changes in lipoproteins, including enrichment of ApoB particles and depletion of ApoA1 and ApoAII [97].

In nephrotic syndrome, a condition frequently associated with progression to advanced kidney disease, PCSK9 expression is markedly upregulated. This leads to excessive LDL receptor degradation, impaired clearance of ApoB-containing particles, and significant elevations in plasma LDL cholesterol [48].

Emerging evidence suggests that PCSK9 inhibitors, by preventing LDL receptor degradation, could substantially improve lipid control in CKD, similarly to statins, and independently from CKD stage; it must be noted that patients with severe CKD or kidney transplant were excluded from main clinical trials [27,98,99]. However, accumulating evidences suggest that PCSK9 inhibitors are effective in the management of dyslipidemia in KTRs with a good tolerability profile [61,100,101]. Thus, PCSK9 could represent both a marker of pathophysiological disturbance and a promising pharmacological target in CKD and transplantation.

### Focus on PCSK9 Inhibitors

Proprotein convertase subtilisin/kexin type 9 monoclonal antibodies (mAbs) have transformed LDL-cholesterol management by preventing PCSK9-mediated degradation of hepatic LDL receptors, thereby markedly enhancing clearance of ApoB-containing particles [102,103,104]. In large cardiovascular outcome trials, evolocumab and alirocumab produced consistent and large LDL-C reductions (roughly 45–55% when added to statin therapy) that translated into significant reductions in major adverse cardiovascular events; these benefits were observed across the range of kidney function represented in those trials [98,102,103]. Pharmacokinetic and pharmacodynamic properties of PCSK9 mAbs make them particularly suitable for patients with renal impairment: as IgG molecules they are primarily metabolized by reticuloendothelial pathways and are not efficiently filtered by the kidney, so renal clearance exerts minimal influence on their disposition and dose adjustment is generally not required [105,106,107,108].

Subgroup and pooled analyses that included participants with mild-to-moderate CKD consistently show preserved LDL-C–lowering efficacy and no signal of renal toxicity, with relative risk reductions for cardiovascular endpoints similar to those seen in individuals with preserved renal function [98,109,110]. A pooled analysis of randomized trials reported comparable LDL-C lowering and safety for alirocumab in participants with and without impaired kidney function (eGFR < 60 mL/min/1.73 m^2^) [109], and the FOURIER subanalysis confirmed consistent LDL-C reductions (~59% with evolocumab) and similar cardiovascular risk reductions across CKD subgroups included in the trial [98]. Importantly, these secondary analyses did not demonstrate meaningful declines in eGFR attributable to PCSK9 therapy during the follow-up intervals studied [98,109].

Evidence for PCSK9 mAb use in advanced CKD, dialysis, and kidney transplant recipients remains limited but grows steadily (Table 2).

Dedicated small studies and real-world series have reported substantial LDL-C reductions and no major nephrotoxicity signals: in a small trial of dialysis patients, biweekly alirocumab produced ~45% LDL-C lowering and ~35% ApoB reduction without evident renal safety issues over 12 weeks [111]. Pharmacokinetic evaluations likewise indicate similar exposure and pharmacodynamic responses to evolocumab in patients with severe renal impairment and in dialysis, supporting dose stability across eGFR strata [108]. Case reports and small case series have described safe administration of PCSK9 mAbs in kidney transplant recipients, without clear evidence of adverse interactions with common immunosuppressants and without observed deterioration in graft function in the short term [101,112,113,114,115]. A recently reported randomized, double-blind study in 197 kidney transplant recipients (evolocumab 140 mg biweekly vs. standard statin therapy) found PCSK9 mAbs to be effective and safe up to 24 months, although larger confirmatory trials are needed to substantiate long-term outcomes [113].

Despite this encouraging signal, important caveats must be emphasized. Major outcome trials largely excluded patients with very advanced renal impairment (e.g., eGFR < 20–30 mL/min/1.73 m^2^) and dialysis patients, limiting direct evidence for the highest-risk groups [116,117]. Thus, conclusions about long-term renal outcomes, effects on proteinuria, graft survival, and hard cardiovascular endpoints in ESRD and transplant cohorts remain provisional and principally informed by small studies, subgroup analyses, and observational data [98,101,108,109,111,113,114,115]. In addition, while PCSK9 mAbs appear to have minimal direct renal toxicity, vigilance is warranted because CKD patients are frequently poly-medicated and possess unique comorbidity burdens that may alter risk–benefit balances in real-world practice [118,119].

In summary, PCSK9 inhibitors provide powerful LDL-C and ApoB lowering and confer cardiovascular benefit in the broader population, with preserved efficacy and reassuring short-term renal safety in patients with mild-to-moderate CKD [52,53,54,55,56,57,58]. Preliminary evidence in dialysis and transplant patients is promising but limited; therefore, dedicated randomized trials and larger real-world registries are needed to define long-term renal safety, optimal monitoring, impact on graft outcomes, and the magnitude of cardiovascular risk reduction in these high-risk subgroups. Given the disproportionate cardiovascular burden among CKD and kidney transplant populations, prioritizing such studies represents an important clinical research need.

## 6. Conclusions

Apolipoproteins and PCSK9 are increasingly recognized as key elements in the complex interplay between lipid metabolism, cardiovascular risk, and renal outcomes in chronic kidney disease and kidney transplantation. Evidence suggests that ApoB, ApoA1, their ratio, and Lp(a) provide more reliable information than conventional lipid markers, not only for assessing cardiovascular risk but also for monitoring CKD progression and post-transplant complications. Genetic factors, such as APOL1 and APOE variants, further influence susceptibility to kidney disease through both lipid-mediated and independent mechanisms, underscoring the multifactorial nature of these conditions.

PCSK9 has emerged as both a biomarker and a therapeutic target, with PCSK9 inhibitors demonstrating high efficacy and safety in the general population. However, robust data in advanced CKD and kidney transplant recipients remain scarce, leaving open important questions regarding their role in this setting.

It must be noted that current evidence on lipid-lowering therapies and the role of PCSK9 inhibitors in renal patients is mostly observational; prospective, interventional studies and randomized-controlled trials are needed in order to establish causality.

Taken together, these findings highlight the need to integrate apolipoproteins and PCSK9 into future research and clinical practice, with the dual aim of refining cardiovascular risk stratification and guiding personalized therapeutic strategies. Addressing these gaps may ultimately improve cardiovascular outcomes, delay disease progression, and extend graft survival in this vulnerable population.

## Figures and Tables

**Table 1:** Lipid profile through CKD and renal replacement therapies.

Lipoproteins	CKD	HD	PD	KT
LDL	⇔	⇔	↑	⇔
HDL	↓	↓	↓	↑
ApoA	↓	↓	↓	⇔
ApoB	↑	↑	↑	↑
Lp(a)	↑	↑	↑	↓
Tryglicerides	↑	↑	↑	↓

CKD, chronic kidney disease; HD, hemodialysis; PD, peritoneal dialysis; KT, kidney transplant; LDL, low-density lipoproteins; HDL, high-density lipoproteins; ApoA, apolipoprotein A; ApoB, apolipoprotein B; Lp(a), lipoprotein (a). ⇔: no variation; ↓: decrease; ↑ increase; Adapted from Barbagallo et al., 2021 [56].

**Table 2:** Effects and safety of PCSK9 monoclonal antibodies across chronic kidney disease stages.

Population	Evidence Type (Examples)	ApoB Effect	Renal Safety/eGFR Effect
Mild-moderate CKD	Large RCTs (FOURIER, ODYSSEY)—subgroup analyses	Marked reduction reported	No clinically meaningful eGFR decline in subgroup analyses
Advanced CKD/ESRD (dialysis)	Small RCTs, pilot studies, real-world cohorts	~30–35% reported in small trial	No major nephrotoxicity signal reported but follow-up short; data sparse
Kidney transplant recipients	Case reports/series; single randomized trial (n = 197)	ApoB reduction reported in small series	No clear graft dysfunction in reports; interactions with immunosuppressants uncommon
Safety/pharmacokinetics across eGFR	Pharmaco studies & reviews	Not reported	mAbs not renally cleared; no dose adjustment generally required

## Data Availability

No new data were created or analyzed in this study. Data sharing is not applicable to this article.

## Abbreviations

The following abbreviations are used in this manuscript:

ApoA1	Apolipoprotein A1
ApoB	Apolipoprotein B
HDL	High density lipoprotein
LDL	Low density lipoprotein
CKD	Chronic Kidney Disease
KTR	Kidney transplant recipient
Lp(a)	Lipoprotein (a)
ESRD	End-stage renal disease
CVD	Cardiovascular disease
CHD	Coronary heart disease
DKD	Diabetic kidney disease
PCSK9	Proprotein convertase subtilisin/kexin type 9
GFR	Glomerular filtration rate
APOL1	Apolipoprotein L1
APOE	Apolipoprotein E
HD	Hemodialysis
CAPD	continuous ambulatory peritoneal dialysis
